# Etiological Factors Affecting Single Versus Multiple Tunneled Hemodialysis Catheter Cuff Extrusions in the Saudi Population

**DOI:** 10.7759/cureus.52906

**Published:** 2024-01-25

**Authors:** Hammad Raza, Muhammad Nauman Hashmi, Muhammad A Khan, Manuel Troncoso, Agamon Christallone, Jawad Alhammouri, Mohamed Hafez, Muhammad Shakeel Khan, Fayez Hejaili

**Affiliations:** 1 Nephrology, South Hemodialysis Center, National Guard Health Affairs, Riyadh, SAU; 2 Nephrology, National Guard Health Affairs, Jeddah, SAU; 3 Medicine, College of Medicine, King Saud Bin Abdulaziz University for Health Sciences, Jeddah, SAU; 4 Research, King Abdullah International Medical Research Center, Jeddah, SAU; 5 Medicine, College of Medicine, King Abdulaziz Medical City, Jeddah, SAU; 6 Nephrology, National Guard Health Affairs, Riyadh, SAU; 7 Nephrology, King Abdulaziz Medical City, Riyadh, SAU

**Keywords:** hypertension, age, perm cath, bmi, recurrent, single, hemodialysis, catheter-related bacteremia, cuff extrusion, tunneled catheter

## Abstract

Objective: To identify underlying factors associated with single versus multiple expulsions of tunneled hemodialysis catheter cuffs and their associated complications.

Materials and methods: A multicenter, five-year retrospective data analysis was conducted on hemodialysis patients with tunneled catheters. Patient data included age, gender, BMI, and associated comorbidities. The type of procedure (new tunnel insertion vs. exchange), exit site infection, and catheter-related bloodstream infection (CRBSI) were also included.

Results: The data of 122 patients was analyzed. Seventy-eight patients had diabetes mellitus, 102 patients had hypertension, and 24 had ischemic heart disease. Fifty-one patients were on antiplatelet therapy, and nine patients were on oral steroids. The access site for 98 patients was the right internal jugular; for 19 patients, it was the left internal jugular; five patients had a femoral dialysis catheter. Patients were grouped into two categories: those with single cuff extrusion episodes and those with multiple cuff-out episodes. Sixty-three patients had one cuff extrusion, and 59 had multiple cuff-out episodes during the study period. Patients who had CRBSI and hypertension and were aged between 61 and 95 had multiple episodes of cuff extrusion and reached statistical significance. Exit-site infection, diabetes mellitus, antiplatelet therapy, gender, catheter site, and BMI had no statistical significance between the two groups.

Conclusion: Tunneled catheter cuff extrusion is a frequent phenomenon. Catheter-related bloodstream infection, hypertension, and age of 61 to 95 years are high-risk factors for multiple episodes of cuff extrusion.

## Introduction

The use of tunneled catheters is common in patients with end-stage renal disease who are starting hemodialysis. In a subset of patients, they are also used for long-term hemodialysis. In North America, up to 33% of patients use tunneled catheters for long-term hemodialysis [1]. This study analyzes factors contributing to the extrusion of the Dacron cuff outside the subcutaneous tunnel, which is defined as the visibility of the tunneled catheter cuff outside the tunnel, either partially or completely. The reported incidence of such a phenomenon in the pediatric population is 25% [2].

Cuff extrusion has also been reported in peritoneal dialysis catheters [3,4]. In a recent study of the adult population, it was observed that the risk of cuff extrusion in dialysis patients increased with obesity, a history of previous cuff extrusion, certain catheter models, and the absence of wing sutures. The incidence of cuff extrusion was reported at 23.8% [5].

The purpose of our study is to identify factors affecting single versus multiple episodes of cuff extrusion in tunneled hemodialysis catheters, as it results in numerous procedures and hospitalizations with a considerable financial impact. It also has the potential to disturb the dialysis schedules of patients and result in undue stress in a population already burdened by chronic illness.

## Materials and methods

A retrospective analysis of the computerized medical records of all patients who had a tunneled hemodialysis catheter was conducted over five years and six months, i.e., from November 2016 until May 2022. This study was approved by the Institutional Review Board of King Abdullah International Medical Research Center, Jeddah, Saudi Arabia (approval no. IRB/2535/22). The patient data was collected from five hemodialysis centers in Saudi Arabia.

All cases of reported permanent catheter cuff extrusion were included in the study. Only Saudi nationals over 18 years of age were included in the study. Baseline data, including age, gender, BMI, and comorbidities (diabetes, ischemic heart disease, congestive cardiac failure, peripheral vascular disease, and hypertension), were recorded. Use of antiplatelets and oral steroids, site of catheter placement, exit site infection, and catheter-related bloodstream infection (CRBSI) were recorded. Procedural details, including the exchange of the catheter over the guide wire or the insertion of a new subcutaneous tunnel, were also analyzed. The primary outcome was to identify factors associated with episodes of multiple cuff-outs in tunneled catheters. All descriptive statistics were analyzed with the use of Fisher's exact test and the chi-squared test. Table 1 shows the age distribution and gender distribution of the studied population, and Table 2 charts their comorbidities and other medical details.

**Table 1:** Age and gender of the studied population

Demographic characteristics		No. of patients (total n = 122)	Percentage
Age	15 to 20 years	1	0.8
	21 to 40 years	22	18
	41 to 60 years	41	33.6
	61 to 95 years	58	47.5
Sex	Male	60	49.2
	Female	62	50.8

**Table 2:** The BMI, comorbidities, and medications in the studied population

Medical characteristics				No. of patients		Percentage
BMI	18-25			33		27
	26-29			21		17.2
	>30			68		55.7
Diabetes mellitus	Yes			78		63.9
	No			44		36.1
Hypertension	Yes			102		83.6
	No			20		16.4
Ischemic heart disease	Yes			24		19.7
	No			98		80.3
Heart failure	Yes			8		6.6
	No			114		93.4
Peripheral vascular disease	Yes			7		5.7
	No			115		94.3
Antiplatelet therapy	Yes			51		41.8
	No			71		58.2
Oral steroids	Yes			9		7.4
	No			113		92.6

## Results

The data of 122 patients was analyzed. Seventy-eight patients were diabetic, 102 were hypertensive, and 24 had ischemic heart disease. The gender distribution was almost equal, and 55.7% of patients had a BMI greater than 30. Fifty-one patients were on antiplatelet therapy, and nine patients were on oral steroids.

Patients were grouped into two categories, i.e., those with single-cuff extrusion episodes and those who had multiple cuff-out episodes. Of the 122 patients enlisted in the study, 63 had one cuff extrusion, and 59 had multiple cuff-out episodes (Table 3). Patients aged between 61 and 95 years who had CRBSI and hypertension had multiple episodes of cuff extrusion and reached statistical significance. However, exit-site infection, diabetes mellitus, antiplatelet therapy, gender, the catheter site, and high BMI had no statistical significance between the two groups.

**Table 3:** Comparison of factors influencing single vs. multiple catheter cuff extrusions † Fisher’s exact test, * Chi-squared test, p-value of <0.05 is considered significant CRBSI: Catheter-related bloodstream infection, IJPC: Internal jugular perm catheter

Characteristics		No. of patients	No. of cuff-out episodes				p-value
			One		Multiple		
			n = 63	%	n = 59	%	
Age	15 to 60 years	64	27	42.2	37	57.8	0.028*
	61 to 95 years	58	36	62.1	22	37.9	
Gender	Male	60	31	51.7	29	48.3	0.995*
	Female	62	32	51.6	30	48.4	
BMI	<30	54	29	53.7	25	46.3	0.684*
	≥30	68	34	50	34	50	
Diabetes mellitus	Yes	78	39	50	39	50	0.630*
	No	44	24	54.5	20	45.5	
Peripheral vascular disease	Yes	7	3	42.9	4	57.1	0.711†
	No	115	60	52.2	55	47.8	
Hypertension	Yes	102	58	56.9	44	43.1	0.009*
	No	20	5	25	15	75	
Heart failure	Yes	8	3	37.5	5	62.5	0.481†
	No	114	60	52.6	54	47.4	
Antiplatelet therapy	Yes	51	24	47.1	27	52.9	0.391*
	No	71	39	54.9	32	45.1	
Current vascular access site	Right IJPC	98	48	49	50	51	0.408†
	Left IJPC	19	11	57.9	8	42.1	
	Femoral	5	4	80	1	20	
New tunnel	0	84	44	52.4	40	47.6	0.807*
	1 or more	38	19	50	19	50	
Exit site infection		89	50	56.2	39	43.8	0.222†
CRBSI		77	49	63.6	28	36.4	0.001†

## Discussion

The hemodialysis patient population is increasing worldwide. Despite many efforts, such as the global Fistula First Initiative, a significant number of patients still start their renal replacement therapy via a tunneled hemodialysis catheter. These catheters have many reported complications, including CRBSI and thrombosis. However, clinical data on cuff extrusion is scarce. Cuff extrusion does happen, and it has multiple adverse effects on the dialysis patient’s management plan. This phenomenon leads to the absence of hemodialysis sessions, increases the risk of CRBSI, and leads to multiple procedures that require hospital visits. This indirectly contributes to a significant escalation in the cost of treatment. The published literature shows that the incidence of cuff extrusion complications can vary greatly, ranging from 0.75% to 19.0% of catheters, or 0.08 to 3.73 per 1000 catheter days [6,7]. Higher rates are reported in the pediatric population (up to 24% of temporary hemodialysis catheters (THDCs) and 2.4 per 1000 catheter days) [8]. A recent study in an adult cohort reported cuff extrusion incidence as 0.79 per 1000 catheter days, and 23.8% (149 of 625) patients experienced this complication. The risk increased with obesity, a history of previous cuff extrusion, certain catheter models, and the absence of wing sutures [5].

In our study, we tried to identify factors that may influence recurrent cuff extrusion in hemodialysis patients using tunneled catheters. Our cohort included patients who were on antiplatelet therapy and oral steroids to see if these medications had any contribution towards cuff extrusion given their anti-inflammatory properties and possible impairment of subcutaneous fibrotic response to Dacron cuff catheters. It is plausible that CRBSI may be a manifestation of open catheter tunnel communication resulting in microbial migration rather than a starting factor, as the incidence of exit site infection between the two groups did not reach statistical significance. Once the Dacron cuff is extruded, the tunneled catheter behaves technically as a non-tunneled catheter. A higher infection rate is well documented in non-tunneled catheters compared to intact-tunneled catheters [9].

Recurrent cuff extrusion was more common in the elderly. This can indirectly be attributed to delayed wound healing, a well-known phenomenon in the elderly population [10]. In the elderly, the healing process is slow, with a reduced inflammatory and proliferative response in addition to a qualitatively different collagen deposition [11]. Age greater than 60 years is an independent risk factor for less frequent closure of chronic wounds [12]. There are other associated conditions in elderly people, such as dementia, a lack of manual dexterity, and visual impairment, which may be contributing to cuff extrusion.

Per our review, the direct association of hypertension with cuff extrusion has not been reported previously. A direct correlation between these two attributes is difficult to explain. Therefore, a future prospective randomized trial may be the right way of determining an answer to this question.

Our study has certain limitations; at the outset, it is a non-randomized retrospective data analysis. Although it is a multi-center study, the population studied belongs to a single ethnic group and environment. The extrapolation of these results to other population groups in other countries may not be suitable. Our study did not confirm the contribution of obesity to recurrent cuff extrusion previously reported. However, the role of the patient’s medication was answered to some extent in our study.

Our study highlights an underreported but clinically significant complication in dialysis patients that has the potential to impair the delivery of good-quality dialysis treatment and increase patient morbidity. This complication puts increased demand on health resources with a higher financial impact. We recommend a larger multicenter, randomized prospective trial to answer this important clinical question.

## Conclusions

Ages 61 to 95, hypertension, and CRBSI are high-risk factors for multiple episodes of cuff extrusion in tunneled hemodialysis catheters. Patients with these risk factors should be prioritized for the early creation of permanent arteriovenous access to minimize patient morbidity and optimize healthcare financial impact. Our study highlights an under-reported but clinically significant complication in dialysis patients that has the potential to impair the delivery of good-quality dialysis treatment and increase patient morbidity. This complication puts increased demand on health resources with a higher financial impact. We recommend a larger multicenter, randomized prospective trial to derive answers that address this. We also recommend considering factors such as visual impairment, dementia, and lack of manual dexterity in catheter cuff extrusion studies, as they may be the reason for this complication in the elderly population.
